# Genes and Pathway Reactions Related to Carotenoid Biosynthesis in Purple Bacteria

**DOI:** 10.3390/biology12101346

**Published:** 2023-10-20

**Authors:** Gerhard Sandmann

**Affiliations:** Biosynthesis Group, Institute for Molecular Biosciences, Fachbereich Biowissenschaften, Goethe Universität Frankfurt, D-60438 Frankfurt, Germany; sandmann@bio.uni-frankfurt.de

**Keywords:** carotenogenic pathways, carotenoid distribution and biosynthesis, enzyme properties, photosynthetic gene clusters, horizontal gene transfer, okenone, reaction mechanisms, spheroidene, spirilloxanthin

## Abstract

**Simple Summary:**

The first-time cloning of genes from a carotenoid pathway was from purple bacteria. The availability of the carotenogenic genes promoted the molecular investigation of the spheroidene and the spirilloxanthin pathway of these bacteria. These genes were applied for heterologous enzyme expression and their characterisation and for genetic pathway complementation. These investigations provided a deeper insight into the course of spheroidene and spirilloxanthin biosynthesis and the similarities between both pathways.

**Abstract:**

In purple bacteria, the genes of the carotenoid pathways are part of photosynthesis gene clusters which were distributed among different species by horizontal gene transfer. Their close organisation facilitated the first-time cloning of carotenogenic genes and promoted the molecular investigation of spheroidene and spirilloxanthin biosynthesis. This review highlights the cloning of the spheroidene and spirilloxanthin pathway genes and presents the current knowledge on the enzymes involved in the carotenoid biosynthesis of purple sulphur and non-sulphur bacteria. Mostly, spheroidene or spirilloxanthin biosynthesis exists in purple non-sulphur bacteria but both pathways operate simultaneously in *Rubrivivax gelatinosus*. In the following years, genes from other bacteria including purple sulphur bacteria with an okenone pathway were cloned. The individual steps were investigated by kinetic studies with heterologously expressed pathway genes which supported the establishment of the reaction mechanisms. In particular, the substrate and product specificities revealed the sequential order of the speroidene and spiriloxanthin pathways as well as their interactions. Information on the enzymes involved revealed that the phytoene desaturase determines the type of pathway by the formation of different products. By selection of mutants with amino acid exchanges in the putative substrate-binding site, the neurosporene-forming phytoene desaturase could be changed into a lycopene-producing enzyme and vice versa. Concerning the oxygen groups in neurosporene and lycopene, the tertiary alcohol group at C1 is formed from water and not by oxygenation, and the C2 or C4 keto groups are inserted differently by an oxygen-dependent or oxygen-independent ketolation reaction, respectively.

## 1. Introduction

Purple bacteria are Gram-negative Proteobacteria defined through their pigmentation. They are distinguished as purple sulphur bacteria and as purple non-sulphur bacteria belonging to different bacterial groups. The purple non-sulphur bacteria are found within the Alphaproteobacteria and the Betaproteobacteria whereas the purple sulphur bacteria are members of the Chromatiaceae within the Gammaproteobacteria [1]. All purple bacteria carry out anoxygenic photosynthesis. The purple non-sulphur bacteria are facultative anaerobes and are able to grow under aerobic conditions by chemotrophy. In contrast, the purple sulphur bacteria growing exclusively phototrophic with hydrogen sulphide as an electron donor are obligate anaerobes [2].

**Scheme 1:**
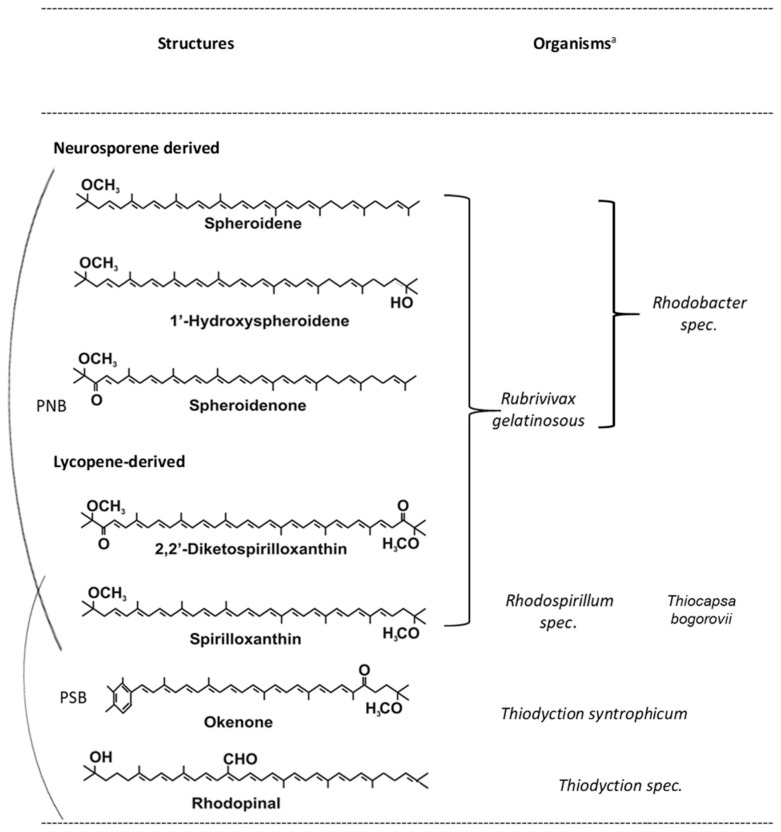
Major carotenoids in purple sulphur (PSB) and nonsulphur (PNB) bacteria ^a^. (^a^ Purple bacteria mentioned in the text). *Rhodobacter spec.* [8]; *Rubrivivax gelatinosous* [10,11,12]; *Rhodospirillum spec.* [8]; *Thiocapsa bogorovii* [17]; *Thiodyction syntrophicum* [13]; *Thiodyction spec.* [15].

The pigmentation of purple bacteria is caused by a combination of bacteriochlorophylls and carotenoids. The carotenoids function in the reaction centres by transferring energy to bacteriochlorophyll in photosynthesis [3]. Their additional role is photoprotection against oxidative degradation [4]. The carotenoid structures vary according to the different proteobacterial groups [5]. Alphaproteobacteria such as *Rhodobacter* (*Rba*.) species (formerly *Rhodopseudomonas* [6]) synthesise neurosporene-derived spheroidene together with 1′-hydroxyspheroidene as the major carotenoids [7]. Others including *Rhodospirillum* (*Rsp.*) species synthesise lycopene-derived spirilloxanthin as the end product of their pathway (see structures in Scheme 1) [8,9]. As a special feature, *Rubrivivax (Rvi.) gelatinosus* of Betaproteobacteria accumulates carotenoids from both pathways [10,11]. Under aerobic conditions, spheroidene is ketolated to spheroidenone by *Rba*. species and spirilloxanthin to its 2-keto and 2,2′-diketo derivatives exclusively by *Rvi. gelatinosus* [12]. Within the purple sulphur bacteria of Gammaproteobacteria, several carotenoid pathways exist. For *Thiodyction* (*Tdc*.) species, the synthesis of okenone (Scheme 1) from γ-carotene [13] but also of rhodopinal (old name warmingone [14]) from lycopene [15] has been reported. Other species from the Chromatiaceae such as *Thiocapsa (Tca.)* alternatively possess a spirilloxanthin pathway [5]. Depending on different species, carotenoids may accumulate as glucosides and glucoside fatty acid esters [16].

The cloning of the photosynthesis gene clusters from purple bacteria provided the genes of their carotenoid pathways [18]. This availability was a breakthrough in the molecular biology of carotenoid biosynthesis and provided support for the biochemical pathway characterisation. It is the focus of this review to highlight the development of carotenoid research with purple sulphur and non-sulphur bacteria presenting the current knowledge from the identification of carotenoid structures to the cloning of the corresponding genes and further on to their application, especially for enzyme expression and the characterisation of individual pathway reactions.

## 2. Carotenogenic Pathways with Related Genes

**Figure 1:**
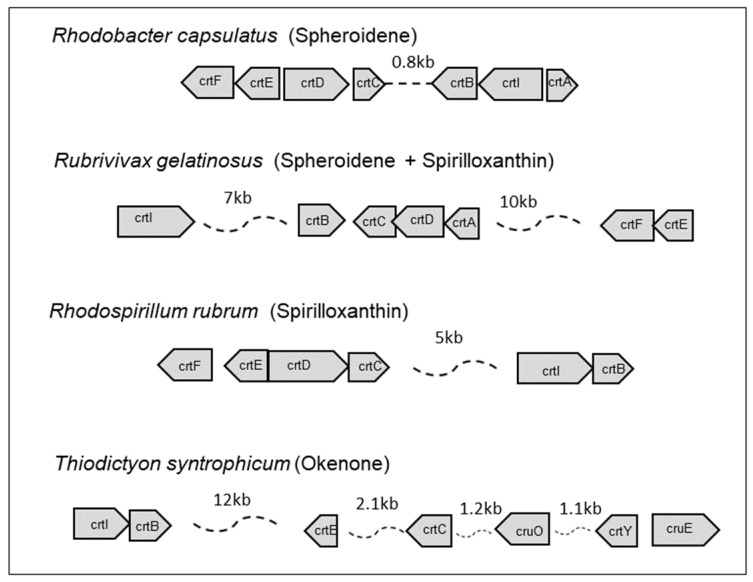
Genome organisation of the structural genes from different carotenoid pathways in purple bacteria (adapted from refs. [13,18,27]).

**Figure 2:**
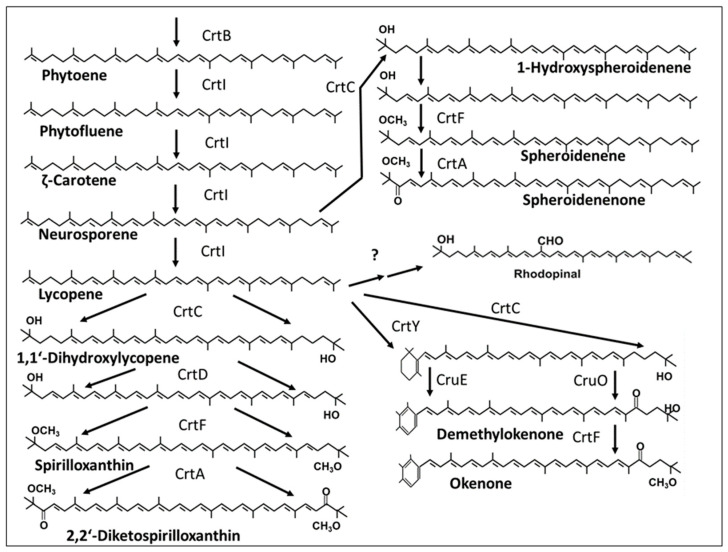
Sequential order of carotenogenic reactions of purple bacteria indicating the enzymes catalysing the individual steps; modifications of both ends of lycopene towards spirilloxanthin is sequential and not simultaneous; 15-cis phytoene is presented in all-trans configuration for simplification; ? indicates that genes and enzymes for the synthesis of rhodopinal from lycopene are still unknown.

The first genes of any carotenoid biosynthesis pathway were obtained and functionally assigned from *Rba. capsulatus*. This work started by gene transfer of the DNA region responsible for pigment biosynthesis and with mutants blocked at different stages of carotenoid biosynthesis [19]. This resulted in a detailed genetic map assigning the loci of the carotenoid biosynthesis *crt* genes and their designation (Figure 1). The *crt* genes in the genome are arranged in two clusters, crtFECD and crtBIA, separated only by the gene of a signal transduction protein TspO. Mutants of Rba. capsulatus with deletions of genes for the pathway from neurosporene to spheroidenone were analysed for the accumulation of carotenoid pathway intermediates [20]. This leads to the following functional gene assignments: *crtC* encoding a hydratase which converts neurosporene to 1-hydroxyneurosporene, *crtD* the gene of a hydroxyneurosporene 3,4-desaturase, *crtF* for a methylase of the 1-hydroxy group of demethylspheroidene and *crtA* the gene fo ra spheroidene 2-ketolase, which is active under (semi)aerobic conditions (Figure 2). Analysis of *crtI*-deficient mutants that accumulated phytoene revealed that this gene encodes a phytoene desaturase [21]. This enzyme is a 3-step desaturase covering the whole sequential course of reactions from phytoene via phytofluene to neurosporene as shown by in vitro reaction [22] confirming that these three reaction steps are catalysed by a single enzyme. The two genes *crtE* and *crtB* were initially wrongly assigned [23,24] and had to be corrected after it had been demonstrated that the orthologues genes from *Erwinia uredovora* encode a geranylgeranyl pyrophosphate synthase and a phytoene synthase, respectively [25]. Also, the order of reactions towards spheroidene [23] had to be revised: enzymatic determination of substrate conversion indicated that after the synthesis of phytoene, the next reaction step is water addition to the C1,2 double bond by CrtC followed by a desaturation reaction catalysed by CrtD forming the C3,4 double bond and by final methylation of the 1-hydroxy group by CrtF [11,26], as illustrated in Figure 2.

Especially under aerobic conditions and strong light, carotenoid biosynthesis is up-regulated to meet the additional demand for antioxidative protection. Studies with *Rba*. species revealed that light [28] and oxygen [29] are the dominating regulatory factors. It has been shown that the *crtI-crtB* operon is transcriptionally up-regulated by oxygen [29]. Upon transition from anaerobic to aerobic growth, *Rba*. species synthesise spheroidenone which is superior to spheroiden as an antioxidant [30]. Higher transcript levels of the spheroidene ketolase gene were also light and oxygen dependent [31]. The gene *tspO* originally assigned as *crtK* [32] is positioned among the *crt* genes [18]. It encodes a regulatory protein that affects *crt* gene expression. This has been shown for *crtA* and *crtI* with a *tspO* deletion mutant. The higher transcript levels for both *crt* genes compared to the wild type indicate a negative regulatory function for TspO in the carotenoid biosynthesis of *Rba*. species [32]. Another positive regulator is RegA [33]. It binds to the promoter region of *crtA* and of the *crtI-crtB* operon. In addition to transcriptional regulation, post-transcriptional regulation of carotenogenic enzymes has been discussed [34].

The end-product of the carotenoid pathway of *Rhodospirillum (Rsp.) rubrum* is spirilloxanthin. The *crt* genes of this purple non-sulphur bacterium were available after the sequencing of its genome later on [35]. As shown in Figure 1, the *crtFECD* gene cluster resembles the same arrangement as in the *Rba. capsulatus* genome [18]. However, the *crtIB* region is much more isolated from the other *crt* genes. It is noticeable that in the genome the same genes as in *Rba. capsulatus* are present, although different carotenoids are formed in both species (Scheme 1). This is due to the broad substrate specificity of caroteneogenic enzymes (see following section). However, a *crtA* gene is missing in the *Rsp. rubrum* genome. This resembles the inability of this bacterium to ketolate spirilloxanthin [36].

*Rvi. gelatinosus* is the only known bacterium with a combined spheroidene and spirilloxanthin pathway which extends to the corresponding 2-keto or 2,2′-diketo derivatives [12] (Figure 2). The genome of this bacterium has been sequenced [37] and the structure of the photosynthetic gene cluster is similar to Alphaproteobacteria [38]. The first localised and identified carotenogenic genes from *Rvi. gelatinosus* were *crtD* and *crtC* [39]. The *crt* genes are scattered in three regions in the genome as clusters *crtADBC* and *crtEF* and as an isolated *crtI* (Figure 1). Obviously, the same genes are involved in both pathways. The succession of the modification reactions starting from lycopene in the spirilloxanthin pathway is the same as for spheroidene synthesis from neurosporene (Figure 2). However, the reactions on the symmetrical lycopene molecule proceed independently at each end [11]. All three purple non-sulphur bacteria, although with different carotenoid pathways, have the same type of genes in common, with the exception of the missing *crtA* gene in *Rsp. rubrum* (Figure 1). The existence of the parallel pathway branches in *Rvi. gelatinosus* demonstrates that CrtC, CrtD, CrtF and CrtA possess a broad substrate specificity, each catalysing the modifications to spheroidene as well as to spirilloxanthin including their ketolation. Phylogenetic analysis of sequences of photosynthesis genes from purple bacteria revealed a high discrepancy between their G+C content and that of their 16S rRNAs indicating that photosynthetic gene clusters including the genes for carotenoid biosynthesis were distributed by lateral gene transfer [40], not only among Alphaproteobacteria but also between them and Betaproteobacteria such as *Rvi. gelatinosus* [41].

The purple sulphur bacterium *Tca. bogorovii* (formerly *Tca. roseopersicina* BBS) possesses a spirilloxanthin pathway [17] as outlined in Figure 2. By mutagenesis, genes in the photosynthetic gene cluster including *crt* genes for the late steps leading to spirilloxanthin synthesis of this bacterium were identified [27]. The Crt proteins exhibited the highest similarities to *Rvi. gelatinosus*. A *crtFECD* gene cluster resembles the genome organisation of *Rba. capsulatus* and *Rsp. rubrum* (Figure 1). However, the linked *crtB* and *crtI* genes are totally isolated from this region.

*Tdc. syntrophicum* is the only purple sulphur bacterium from which the genes of the okenone pathway are known and functionally assigned [13]. These genes of the carotenoid pathway appear in two distant groups in the genome: *crtIB* and *crtEC-cruSO-crtYU*. The gene *crtF*, which is absent from both clusters, is positioned in an unlinked region of the genome. Some of the carotenogenic genes of the okenone pathway resemble those found in purple non-sulphur bacteria where they are responsible for the modifications of the acyclic ends of neurosporene and lycopene in the formation of spheroidene and spirilloxanthin. Cyclisation of one end of lycopene is catalysed by the CrtY-type lycopene cyclase yielding a β-ring which is further converted to the aromatic χ-ring of okenone (Figure 2) by the product of a gene assigned as *crtU* in ref. [13]. However, a *crtU* gene was first cloned from isorenieratene-synthesising *Streptomyces griseus*, where it is involved in the formation of an aromatic φ-ring [42]. According to its function, the gene involved in χ-ring synthesis in *Tdc. syntrophicum* resembles the *cruE* gene responsible for the aromatic χ-ring in cyanobacterial synechoxanthin synthesis [43]. Two other novel carotenogenic genes have been detected in the *Tdc. syntrophicum* genome, *cruS* and *cruO*, which are distantly related to *crtD* 130]. It has been demonstrated that *cruO* encodes a 4-ketolase which is specific for okenone biosynthesis (Figure 2). Upon genetic complementation with *cruS*, ketolation of spheroidene at C2 was observed. Neither is this reaction part of the okenone pathway nor was a 2-ketocarotenoid in *Tdc. syntrophicum* detectable [13]. The relevance of *cruS* in the pathway remains obscure.

The function of the carotenogenic genes of *Tdc. syntrophicum* was elucidated by genetic complementation first introduced for the determination of the number of desaturation steps catalysed by CrtI starting from phytoene [13]. This procedure involves the co-transformation of a host with the gene of an enzyme to be analysed together with a substrate-providing plasmid following analysis of the reaction product.

Among purple bacteria, *Rba. sphaeroides* has been developed as a cell factory for the heterologous production of metabolites [44], and after the identification of its *crt* genes on the photosynthesis gene cluster [45], this bacterium was used for the synthesis of carotenoids. By using the gene of a 4-step phytoene desaturase to replace the endogenous gene, lycopene has been accumulated [46]. In a more complex metabolic engineering approach, *crtC* was inactivated and the competitive pentose phosphate pathway was blocked [47]. For the synthesis of β-carotene, a bacterial *crtY* lycopene cyclase gene with selected promoter in addition to the gene of a 4-step phytoene desaturase was engineered into *Rba. sphaeroides*. Together with an enhancement of precursor supply by overexpressing the gene of a limiting reaction and by blocking competing reactions, high levels of β-carotene could be produced [48].

## 3. Reactions and Properties of Enzymes of the Carotenogenic Pathways of Purple Bacteria

Enzymes of secondary metabolism such as the terpenoid pathway including carotenoid biosynthesis are generally present in low abundance. Furthermore, most carotenogenic enzymes are membrane-bound and can be extracted only after solubilisation which is accompanied by loss of activity. This hampers the isolation and characterisation of these enzymes. The availability of carotenogenic genes opens the possibility of heterologous enzyme expression in suitable quantities. The potential of this strategy and its application for enzyme provision, purification and assessment of catalytic properties has been pointed out [22].

### 3.1. CrtE and CrtB, Comparison to Other Bacteria 

The reaction catalysed by CrtE provides geranylgeranyl pyrophosphate (GGPP) [49] for the synthesis of phytoene, the first compound in the specific carotenoid pathway, which is catalysed by CrtB [25] as outlined in Figure 2. Both enzymes have not been characterised by a purple bacterium but due to the high similarities to the genes from *E. uredovora* [24], equivalent properties can be assumed. CrtE from *E. uredovora* is a classical prenyl transferase using the allylic farnesyl pyrophosphate or geranyl pyrophosphate as substrates but has a very low affinity for dimethyl allyl pyrophosphate [50]. This is in contrast to other bacterial GGPP synthases [51,52]. Nevertheless, they all share the same product specificity with the formation of GGPP as the only reaction product regardless of the allylic substrate. The phytoene synthase CrtB from *E. uredovora* which converts two molecules of GGPP to 15-cis phytoene is an ATP- and Mn^2+^- or Mg^2+^-dependent enzyme which is inhibited by phosphate ions and squalestatin [53]. 

### 3.2. CrtI Determines the Pathways towards the End Products

Phytoene desaturases CrtI are common to carotenogenic bacteria. All enzymes exhibit the same substrate specificity but may differ in the number of desaturation steps [54], which in purple bacteria determines the type of carotenoid pathways. The 3-step phytoene desaturase from *Rba. capsulatus* has been heterologously expressed, purified and then characterised [22]. In vitro, it catalyses the whole desaturation sequence from phytoene via phytopfluene and ζ-carotene to neurosporene but not further on. This is a special feature of the desaturase from *Rba*. species and related members from the family of Rhodobacteraceae. The affinity of the enzyme for ζ-carotene was more than twice as high as for phytoene. This explains the effective conversion of ζ-carotene to neurosporene without the accumulation of ζ-carotene or related products. The cofactor in the desaturation steps of CrtI from *Rba. capsulatus* was FAD as a hydrogen acceptor. In *Rvi. gelatinosus*, CrtI is responsible for the formation not only of neurosporene but also for an additional desaturation step to lycopene [10]. Subsequently in this bacterium, both carotenes are the starting points for the spheroidene and the spirilloxanthin pathway, respectively. Thus, the degree of desaturation at C7,8 exclusively determines the formation of the different pathways, since all the enzymes catalysing the following modification steps (Figure 2) do not select between the neurosporene or lycopene backbone and react with both. This is due to the broad substrate specificity of carotenogenic enzymes since only the end of the carotenoid molecule is sufficient for binding to the substrate recognition sites of the enzymes (see Table 1 for examples). Other non-sulphur bacteria such as *Rsp. rubrum* possess a CrtI which mediates the formation of lycopene as the exclusive desaturation product [55]. This type of 4-step desaturase has been enzymatically characterised from *E. uredovora* [56]. It is closely related to the enzyme from *Rba. capsulatus* both using FAD as the hydrogen acceptor. Unlike as discussed earlier [57], the desaturation to neurosporene and then further on to lycopene is catalysed by a single enzyme and not by two successively acting desaturases. This is supported by the presence of only one *crtI* gene in all genomes of purple bacteria [18].

The reaction mechanism of all types of CrtI desaturases is similar. It starts with a hydride transfer from an allylic carbon resulting in the formation of an allylic carbocation (Table 2, **a**) [54]. The next step is proton abstraction from an adjacent carbon atom. The CrtI-type of desaturation which inserts a new double and additionally integrates an isolated double into the polyene system is thermodynamically favoured due to the extension of the conjugated double bond system. A mechanism for the isomerisation of the C15,15′ cis-double bond of phytoene to all-trans during the desaturation process has been proposed [54]. All CrtI enzymes belong to the flavoprotein disulphide oxido-reductase superfamily sharing similar structural features in different regions, especially a βαβ fold at the N-terminus. This specific domain where FAD is non-covalently bound for catalytic interaction with the enzyme was identified in CrtI [25], together with the active site containing the cysteine pair of the redox centre [60].

It has been shown that the extent of desaturation steps by CrtI is determined in two ways, by alteration of enzyme kinetics or by mutations in the enzyme [42]. Mutants of the *crtI* gene were generated to alter the catalytic properties of this desaturase in two different directions. CrtI mutants from *Rba. spheroides* changed from neurosporene to lycopene production [61]. Additionally, two sets of CrtI mutants with altered properties were obtained from *Rvi. gelatinosus* [62]. One type showed much lower or almost zero lycopene-synthesising activity. In another mutant, the affinity for neurosporene as substrate desaturation was substantially increased, resulting in the domination of lycopene over neurosporene formation. This particular mutant carried a single amino acid exchange in a conserved hydrophobic region which is regarded as the binding site for carotenes. This corresponds to several CrtI mutants from *Rba. spheroides* with acquired lycopene synthesis caused by amino acid exchanges within the putative substrate-binding site or the FAD-binding region [61]. Most of these CrtI mutations affected the protein structure which altered substrate binding which affects the affinities for individual carotenes during the desaturation process.

**Table 2:** Reaction mechanisms of enzymes related to carotenogenesis in purple bacteria.

Enzymes	Reactions		References
**a.** Crtl-related enzymes	Formations of carbocation:	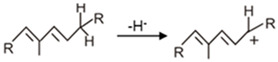	[54]
	Stabilisation reactions:		
Crtl	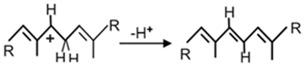		[54]
CrtD	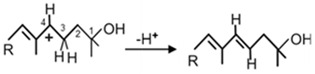		[54]
CruH (CrtO ^a^)	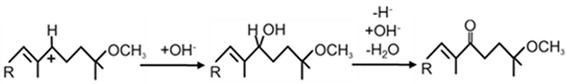		[63]
**b.** CrtY	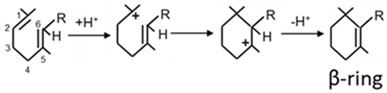		[64]
**c.** CruE-CrtU	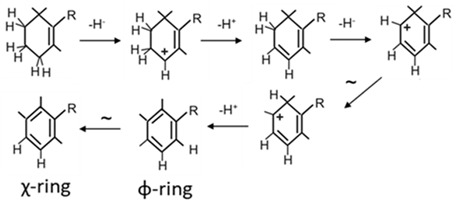		[64,65]
**d.** CrtA	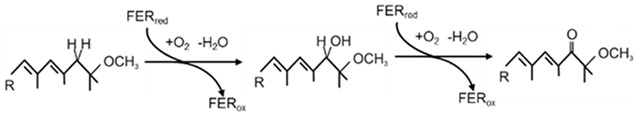		[59]

Relevant hydrogen groups are accentuated; R1 and R2 in the CrtI react ion indicates the remaining residue of the C40 carotenoid structure according to Figure 2; ^a^ mechanisms proposed in analogy to closely related CrtO.

As judged from the distribution of dominating lycopene over neurosporene pathways in bacteria, it is most likely that the *crtI* gene in Rhodobacteraceae evolved from a *crtI* gene of a lycopene-synthesising desaturase. *Rvi. gelatinosus*, in which the activity of the lycopene-derived spirilloxanthin, in addition to the neurosporene formation, is still retained, can be considered as a missing link between purple bacteria with either an exclusive spheroidene or spirilloxanthin pathway. However, a bacterium with an exclusive spheroidene pathway could have only survived under aerobic conditions with a co-acquirement of the *crtA* gene [66]. In the presence of oxygen, the CrtA ketolase is up-regulated and then modifies spheroidene to spheroidenone (Figure 2). The resulting extended conjugated double-bond system in this keto carotenoid has a lower energy level of the triplet state than in spheroidene [67]. Unlike spheroidene, this makes spheroidenone an effective photoprotective quencher of singlet oxygen, which is essential to protect the photosynthesis apparatus of Rhodobacteraceae under aerobic conditions.

### 3.3. CrtC for Anaerobic Formation of a Tertiary Alcohol Group

CrtC catalyses the addition of water to the C1,2 double bond of neurosporene or lycopene. Another 1,2-hydratase CruF from Cyanobacteria is completely unrelated to CrtC although the reaction with the formation of the tertiary hydroxyl group at C1 is the same [68]. The addition of water to form the 1-hydroxyl group in *Rba sphaeroides* was confirmed by labelling studies [69]. After heterologous expression, the CrtC hydratases have been isolated and purified from different purple bacteria, *Rba. capsulatus*, *Rvi. gelatinosus* and *Tca. bogorovii* [26,70]. Analysis of their catalytic properties demonstrated that the reactions are independent of any cofactor. Enzyme kinetic studies with CrtC from *Rba. capsulatus* and *Rvi. gelatinosus* compared the substrate specificity of the enzymes from both species within pathways leading either exclusively to spheroidene or additionally to spirilloxanthin. Both enzymes catalyse the formation of a 1-hydroxyl group of neurosporene and lycopene. However, only the enzyme from *Rvi. gelatinosus* could insert a second 1′-hydroxyl group either into hydroxylycopene or spheroidene converting the latter into 1′-hydroxyspheroidene (Table 1). In contrast, the hydratase originating from *Rba. capsulatus* was not active enough to convert both carotenoids as substrates in the in vitro reaction [26]. Nevertheless, 1‘-hydroxyspheroidene is present in *Rba. capsulatus*, however only in trace amounts in the investigated strain [20]. The 1′-hydroxyl group may be inserted at an earlier stage of the pathway for example in a reaction yielding 1′-hydroxy-1′,2′,3,4-tetrahydrospheroidene as an intermediate which has been identified in *Rsp. Rubrum* [71]. The enzymatic properties of CrtC hydratase from the purple sulphur bacterium *Tca. Bogorovii* with an exclusive spirilloxanthin pathway resembled those of the *Rvi. Gelatinosus* enzyme [70]. The substrate specificity was similar in accepting neurosporene and lycopene as substrates as well as 1-hydroxylycopene.

The characteristics of CrtC from all investigated purple bacteria confirm a reaction mechanism that starts with the addition of a proton to the C1,2 double bond in a similar way as in the starter reaction of lycopene cyclisation (Table 2, **b**), resulting in a carbocation at C1 which is then neutralised by a reaction with a hydroxyl group yielding the 1-hydroxyl moiety in the different carotenoid pathways of purple bacteria (Figure 2). The active site for this acid-base type reaction is expected at a region of four specific amino acids which were identified by mutation analysis [72].

### 3.4. CrtD, a 3,4-Desaturase of the CrtI-Family

CrtD is a 1-hydroxy-ψ-carotene 3,4-desaturase closely related to CrtI [73] with a similar desaturation mechanism (Table 2, **a**). In analogy to the catalysis by CrtI, hydrogen abstraction from C4 and stabilisation of the resulting allylic carbocation by proton leaving from C3 forms the C3,4 double bond. The principle difference to CrtI is its specificity for the substrate. The purified CrtD enzymes from *Rba*. *capsulatus* [74] and *Rvi. gelatinosus* [11] modify the 3,4-dihydro part of a 1-hydroxy-ψ-end group to a 3,4 double bond (Table 1). While 1-hydroxyneuropsorene and 1-hydroxylycopene are converted by CrtD, the corresponding O-methylated derivatives are not desaturated indicating that an unsubstituted 1-hydroxyl group is essential for substrate recognition [11,74]. Subsequent formation of both hydroxyl groups at C1 and C1′ of lycopene before desaturation of C3,4 results in a dead end in the spirilloxanthin pathway of *Rvi. gelatinosus*. Enzyme kinetics of CrtD from this purple bacterium reveals a higher affinity for 1-hydroxyneurosporene than for 1-hydroxylycopene which coincides with the dominance of spheroidene over spirilloxanthin synthesis [10,11]. For CrtD neither from *Rba*. *capsulatus* nor from *Rvi. gelatinosus*, the genuine hydrogen acceptor for desaturation could be determined due to the interference of oxygen in the in vitro reaction [11,74].

### 3.5. CrtF, a Conserved Methyltransferase

CrtF from purple bacteria is a highly conserved O-methyltransferase [75]. It catalyses the final steps in the pathways to spheroidene and spirilloxanthin [11]. Growth of *Rba*. *capsulatus* in the presence of ^14^C-methylmethionine resulted in the labelling of the O-methyl group of methoxyneurosporene [20,76]. The purified methyltransferase from *Rba*. *capsulatus* uses S-adenosylmethionine as a cofactor [54]. Due to the broad substrate specificity, CrtF converts acyclic 1-HO-carotenoids with a 5,6-double bond in conjugation to the central polyene chain in the spheroidene pathway. But this enzyme is also able to methylate intermediates of the spirilloxanthin pathway (Figure 2) which exists in other purple bacteria (Figure 2). The lack of the 7,8-double bond as in 1-hydroxy-ζ-carotene prevents further methylation by CrtF. This explains why 1′-hydroxyspheroidene accumulates instead of a 1′-methoxy end product.

### 3.6. CrtY, the Dominating Lycopene Cyclase in Bacteria

Within the bacteria, four different types of lycopene cyclases for the formation of a β-ionone ring exist [77]. The most widespread is CrtY, which is one of the enzymes in the pathway to okenone in *Tdc. syntrophicum* converting lycopene into γ-carotene [13]. Enzymatic data on this enzyme from purple sulphur bacteria are not. 

The reaction of lycopene cyclisation starts with proton addition to the C1,2 double bond [78]. The carbocation at C1 which is favoured due to the two methyl groups then adds to the C5,6 double bond to form a 6-membered ring (Table 2, **b**). Finally, the carbocation at C5 is stabilised by proton loss from C6 regaining the C5,6 double bond. Proton addition and abstraction to and from the indicated carbons have been demonstrated by isotope labelling [79,80].

### 3.7. CruE versus CrtU for the Formation of Different Aryl End Groups

The gene for the desaturation of the β-ring to the aromatic χ-ring with its 1,2,3-trimethyl substituents (Table 2, **c**) has been cloned from *Tdc. syntrophicum* [13]. It shows similarity to the *cruE* gene from cyanobacteria which encodes an enzyme for the synthesis of this χ-ring [43]. Despite the similarity to *crtU*, the gene for the synthesis of φ-rings with 1,2,5-trimethyl substituents [42] is even higher compared to *cruE* from green sulphur bacteria [81], the reaction catalysed by this *Tdc. syntrophicum* desaturase results in the formation of a χ-ring of okenone. Consequently, this desaturase gene should be and will be in this text referred to as *cruE*. The close relationship of *crtU* and *cruE* is reflected by the partially common mechanisms of the reactions catalyses by both enzymes. 

The reaction by CruE starts in analogy to other desaturations with an allylic carbocation at C4 of the β-ring and the formation of a C3,4 double bond. A second desaturation yielding the additional C1,2 double bond coincides with a methyl transfer from C1 to C2 resulting in the intermediary formation of the 1,2,5-trimethyl-substituted aromatic φ-group. The consecutive formation of the χ-ring with methyl groups at positions 1, 2 and 3 includes an additional shift of the 5-methyl. An isomerisation mechanism for this second migration from C5 to C3 which changes the φ- into an χ-ring has been proposed to occur through a prismane structure [65]. In cyanobacteria that synthesise synechoxanthin with χ-end groups, isorenieratene with φ-end groups was also detected to a certain extent as a possible intermediate [82]. This finding supports a reaction mechanism of catalysis by CruE via the 1,2,3-trimethyl intermediate, as outlined above.

### 3.8. CrtA and CruO: Oxygen-Dependent and Oxygen-Independent Ketolases

Hydroxylation as part of the formation of keto moieties can be catalysed by oxygen-dependent oxygenases or oxygen-independent enzymes, the latter using water as the source of the keto group. Oxygen-dependent monooxygenases carry out hydroxylation with molecular oxygen retaining one oxygen atom of O_2_ to be reduced to water [83]. One type of monooxygenase is the P450 enzymes which use electrons from NAD(P)H as an electron donor [84]. In contrast to monooxygenases, the dioxygenase reaction integrates both oxygen atoms into the substrate(s) [85].

Different types of ketolation reactions can be found in the bacterial carotenoid pathway of non-purple and purple bacteria. CruO from purple sulphur bacteria is a 4-ketolase that forms a keto group at position 4 in the okenone pathway of anaerobic-growing purple sulphur bacteria [13]. It shares a high similarity with CrtO and is consequently a member of the CrtI family. Therefore, in analogy to CrtO, the same reaction mechanism should apply (Table 2, **a**) in a similar way as proposed by Goodwin [64]. The allylic C4 carbocation formed by hydrogen transfer as the starting reaction of CrtI-type catalysis reacts with a hydroxyl group [63]. The repetition of this reaction results in a second hydroxyl group at C4, which after water elimination forms the 4-keto moiety of okenone. In this pathway, CruO replaces the evolutionary and mechanistically related CrtD by the formation of a 4-keto group instead of the C3,4 double bond as in the spheroidene and spirilloxanthin pathways.

The ketolases CrtA is distinct and specific for purple non-sulphur bacteria. The purified enzymes from *Rba. capsulatus* as well as *Rvi. gelatinosus* require molecular oxygen and reduced ferredoxin for the catalytic activity in a monooxygenase manner [59]. This is indicated by the mechanism outlined in Table 2, **d**. Two subsequent hydroxylation steps at C2 each yielding a hydroxyl group and a molecule of water are followed by the loss of a water molecule from the two hydroxyl groups which results in the formation of the C2 keto group. This mechanism is distinct from the catalysis by CrtW, a different ketolase introducing a keto group at C4 of carotenoid β-rings with ascorbate as an electron donor found in a variety of bacterial groups [86]. CrtW belongs to the dioxygenase type which uses 2-oxoglutarate as co-substrate [85]. In contrast to other monooxygenases, CrtA is not a P450 enzyme but instead possesses a 5-coordinated heme at the active site [87]. The substrate specificities of CrtA enzymes for products from the spheroidene and the spirilloxanthin pathway from both *Rba*. species are very broad, even for the enzyme from *Rba. capsulatus* which ketolates spirilloxanthin but lacks its biosynthesis. Formation of diketospirilloxanthin involves two consecutive ketolation steps (Figure 2) in which the ketolation at one acyclic end is independent of the ketolation of the other [88]. The affinity for ketospirilloxanthin in the second step was higher than for spirilloxanthin [59] which corresponds to the dominance of the diketo end product.

Phylogenetic considerations indicate that *crtA* originates from the closely related *crtA-OH* gene from a *Flavobacterium* which encodes a hydroxylase [89]. This gene evolved towards a duplicated hydroxylation step at C2, finally resulting in the ketolating property of CrtA (Table 2, **d**) followed by horizontal gene transfer from Bacteroidetes into purple non-sulphur bacteria [66].

## 4. Conclusions

Purple bacteria are prominent candidates to study the biosynthesis pathways, especially of acyclic carotenoids. The genes of carotenoid biosynthesis are part of closely related photosynthesis gene clusters which were distributed among purple bacteria by horizontal gene transfer. The cloning of these genes from purple bacteria was a pioneering first step that opened the fields of molecular biology and boosted the biochemical elucidation of carotenoid biosynthesis. The available genes provided novel tools for the characterisation and molecular investigation of carotenoid pathways not only for purple bacteria but also for other pro- and eukaryotic organisms. This allowed targeted mutations and heterologous gene expression for enzyme characterisation, including genetic complementation, to broaden our understanding of the spheroidene and spirilloxanthin pathways. Based on the catalytic properties of some of the heterologously expressed enzymes, the underlying reaction mechanisms of individual steps in the pathways have been established. Diverse oxygen-dependent or independent reactions are involved in the formation of hydroxyl and corresponding keto groups which exist among the purple bacteria. The substrate preference of the pathway enzymes revealed the order of the modifications of the carotenoids leading to spheroidene and spirilloxanthin formation. The substrate specificities of some enzymes also explain the presence of side products that cannot be further metabolised such as 3,4-dihydrospheroidene [5] and is the reason why 1′-hydroxyspheroidene is accumulated instead of a 1′-methoxy end product in the spheroidene pathway. Metabolite flow into the spheroidene or spirilloxathin pathway branches is determined by the product specificity of CrtI catalysing the formation of neurosporene and/or lycopene, respectively. The modifications of the ψ-end group to spheroidene and both ψ-ends of lycopene to spiriloxanthin are the same and are catalysed by enzymes with similar catalytic properties. This is also the case for the conversions at the acyclic end of okenone, with the exception of the missing 3,4-desaturation step, which is replaced by 4-ketolation. The corresponding CruO 4-ketolase is phylogenetically related to the 3,4-desaturase CrtD, both belonging to the CrtI family.

In recent years, investigation of carotenoid biosynthesis pathways in purple bacteria was neglected. For example, we still know nothing about the genes and enzymes of the individual steps to rhodopinal and about glycosylation reactions esterification with fatty acids [5]. Therefore, this review may revive the interest in the investigation of the biosynthesis of acyclic carotenoids for which purple bacteria are an attractive system.

## Figures and Tables

**Table 1:** Carotenoid substrates converted by enzymes from purple non-sulphur bacteria.

1,2-Hydratase CrtC from *Rvi. gelatinosus* [26]:
Neurosporene ^a^, Lycopene ^a^, 1-HO-Neurosporene, 1-HO-Lycopene,
Demethylspheroidene, 1-HO-3,4-Didehydrolycopene, Spheroidene
3,4-Desaturase CrtD from *Rvi. gelatinosus* [11]:
1-HO-ζ-Carotene, 1-HO-γ-Carotene, 1-HO-Neurosporene,
1,1′-(HO)_2_-Neurosporene, 1-HO-Lycopene, 1,1′-(HO)_2_-3,4-Didehydrolycopene,
1-HO-3′,4′-Didehydrolycopene, 1-CH_3_HO-1′-HO-3,4-Didehydrolycopene
1-HO-Methylase CrtF from *Rba. capsulatus* [58]:
1-HO-Neurosporene, 1,1′-(HO)2-Neurosporene, Spheroidene, 1‘-HO-Demethylspheroidene,
1-HO-Lycopene, 1,1′-(HO)_2_-Lycopene, 1-HO-3,4-Didehydrolycope,
1,1′-(HO)_2_-3,4-Didehydrolycope, 1,1′-(HO)_2_-3,4,3′,4′-Tetradehydrolycope
2-Ketolases CrtA from *Rvi. gelatinosus* [59]:
Spheroidene, 1-HO-Spheroidene, Spirilloxanthin, 2-Ketospirilloxanthin

Phytoene desaturase specificity is restricted to the substrates indicated in the reaction in Figure 2 [22]; ^a^ the only substrates for *Rba. capsulatus* CrtC.

## Data Availability

No new data were created or analysed in this study. Data sharing does not apply to this article.
